# Inverted internal limiting membrane flap technique versus internal limiting membrane removal in large macular hole with different hole edge configurations: a comparative study

**DOI:** 10.3389/fmed.2026.1800139

**Published:** 2026-04-13

**Authors:** Xiang-Yue Zheng, De-Jin Cheng, Xiao-Yi Yu, Ze-Feng Xiao

**Affiliations:** 1 Guangzhou University of Chinese Medicine, Guangzhou, China; 2 Department of Ophthalmology, Wuhan No.1 Hospital, Wuhan, China; 3 Department of Nephrology, The Central Hospital of Wuhan, Wuhan, China; 4 Department of Ophthalmology, Guangzhou Aier Eye Hospital, Guangzhou, China

**Keywords:** internal limiting membrane flap, internal limiting membrane removal, intraoperative optical coherence tomography, macular holes, retina microstructure, visual outcomes

## Abstract

**Purpose:**

To compare the anatomical and functional outcomes of the inverted internal limiting membrane (ILM) flap technique and the complete ILM removal in the treatment of large macular hole (MH) > 400 μm with or without Hole-door structures.

**Methods:**

This retrospective study analyzed 56 patients with MH > 400 μm. Based on hole edge morphology evaluated by archived intraoperative optical coherence tomography (iOCT) images, patients were divided into the Hole-door group (Group 1) and the Negative group (Group 2). Within each group, patients were further classified into two subgroups based on the surgical technique performed: complete ILM removal (Group 1-a, 2-a) and visco-assisted ILM flap (Group 1-b, 2-b). External limiting membrane (ELM) and ellipsoid zone (EZ) restoration and best-corrected visual acuity (BCVA) were assessed at 3 and 6 months post-surgery.

**Results:**

Correlation analysis found no significant link between preoperative MHD and postoperative outcomes. The Hole-door group showed better vision, ELM, and EZ recovery than the Negative group, regardless of ILM removal or visco-assisted ILM flap (*P* < 0.05). In evaluating the prognoses between both techniques for macular holes with or without Hole-door structures, at 6 months, BCVA and ELM/EZ restoration were similar in Groups 1-a and 1-b (*P* > 0.05), but at 3 months, Group 1-b showed better results (*P* < 0.05). In the negative subgroups, Group 2-b outperformed Group 2-a in BCVA and ELM and EZ restoration at 6 months (*P* < 0.05) and in BCVA and ELM restoration at 3 months, though not in EZ restoration (*P*<0.05) Furthermore, functional and anatomical outcomes at 3 months showed significant correlation with the 6-month results, despite the ongoing microstructural improvements identified between these two time points.

**Conclusion:**

Large macular holes with Hole-door structures have a better prognosis than those without. In these cases, BCVA and retinal microstructure restoration are similar between ILM removal and ILM flap surgery, but the ILM flap accelerates recovery.

## 1 Introduction

A macular hole (MH) is a retinal defect that originates at the internal limiting membrane (ILM) and extends to, but does not include, the retinal pigment epithelium (RPE) within the foveolar region. Primary MH, which arises from vitreomacular tractions—either tangential or anteroposterior—is predominantly observed in the elderly population, with a prevalence of 0.2–0.8% in the general population (1).

In macular hole surgery, intraoperative optical coherence tomography (iOCT) provides essential real-time feedback and guidance during the procedure. The implementation of iOCT not only enhances surgical success rates but also facilitates improved recovery of postoperative visual function (2–4). Findings from the DISCOVER study indicate that iOCT offers valuable insights during macular hole repair surgery, aiding in the confirmation of vitreomacular traction release and the identification of hidden residual membranes. In 51% of cases, iOCT provided actionable feedback, and in 12% of cases, iOCT data directly influenced surgical decisions. These findings underscore the significance of iOCT in macular hole surgery, as it not only improves surgical outcomes but also substantially enhances postoperative visual function (5).

Recent advancements in surgical approaches for macular holes, particularly in managing large-diameter and refractory cases, have been remarkable. Pars-plana vitrectomy (PPV) with ILM removal and gas tamponade is the conventional treatment for MH, achieving about a 90% closure rate. Nonetheless, large MHs (> 400 μm), chronic MHs, and secondary MHs resulting from trauma or other conditions frequently exhibit suboptimal outcomes (6, 7). In 2010, Michalewska et al. introduced the inverted ILM flap technique, which enhanced both visual acuity and closure rates for large idiopathic and myopic MHs (8). A study conducted by Rizzo et al. demonstrated closure rates of 95.6% for idiopathic MHs greater than 400 μm and 88.4% for myopic MHs utilizing this technique (9).

The restoration of visual acuity following macular hole surgery is affected by various factors, including the size and morphology of preoperative macular holes, the duration of the condition (10, 11), the diameter of the IS/OS junction defect (12), the recovery of the external limiting membrane (ELM) and ellipsoid zone (EZ) (13, 14), and the reestablishment of the Cones Outer Segment Terminal Line (COST line) (15). Comprehensive research into these factors can enhance our ability to predict and improve visual recovery outcomes post-surgery. Additionally, the morphology of macular hole edges is considered a critical predictor of postoperative retinal microstructure recovery and functional outcomes. Studies utilizing iOCT have demonstrated that different edge morphologies can be used to forecast postoperative retinal microstructure recovery, identifying the Hole-door structure as an indicator of favorable postoperative visual recovery (16). However, the role of macular hole edge morphology in guiding the selection of surgical strategies remains insufficiently analyzed.

In this study, iOCT was utilized following ILM peeling during vitrectomy for large macular holes to elucidate the morphological alterations at the MH edges. Through iOCT imaging, we characterized the morphology of the hole edge, specifically noting the presence or absence of a Hole-door structure post-ILM peeling. Participants with or without Hole-door structure were both stratified into two groups: one group underwent ILM removal, while the other received an ILM flap. Subsequent analyses concentrated on the restoration of retinal microstructure and the visual outcomes following surgery.

## 2 Materials and methods

### 2.1 Patients

This study was a retrospective, consecutive case series of patients with large idiopathic macular holes (MHD > 400 μm) who underwent vitrectomy in the Department of Ophthalmology at Wuhan No. 1 Hospital between July 2022 and December 2024. The exclusion criteria were history of penetrating trauma, high myopia, previous vitreoretinal surgery, and final follow-up less than 6 months, and eyes that underwent PPV without iOCT.

According to the morphological characteristics of the hole edge observed through iOCT after internal limiting membrane (ILM) peeling, all patients were divided into two groups. The Hole-door group (Group 1) was defined by the presence of vertical pillars of tissue extending into the vitreous cavity from the hole edges, while the Negative group (Group 2) showed no such features.

**FIGURE 1:**
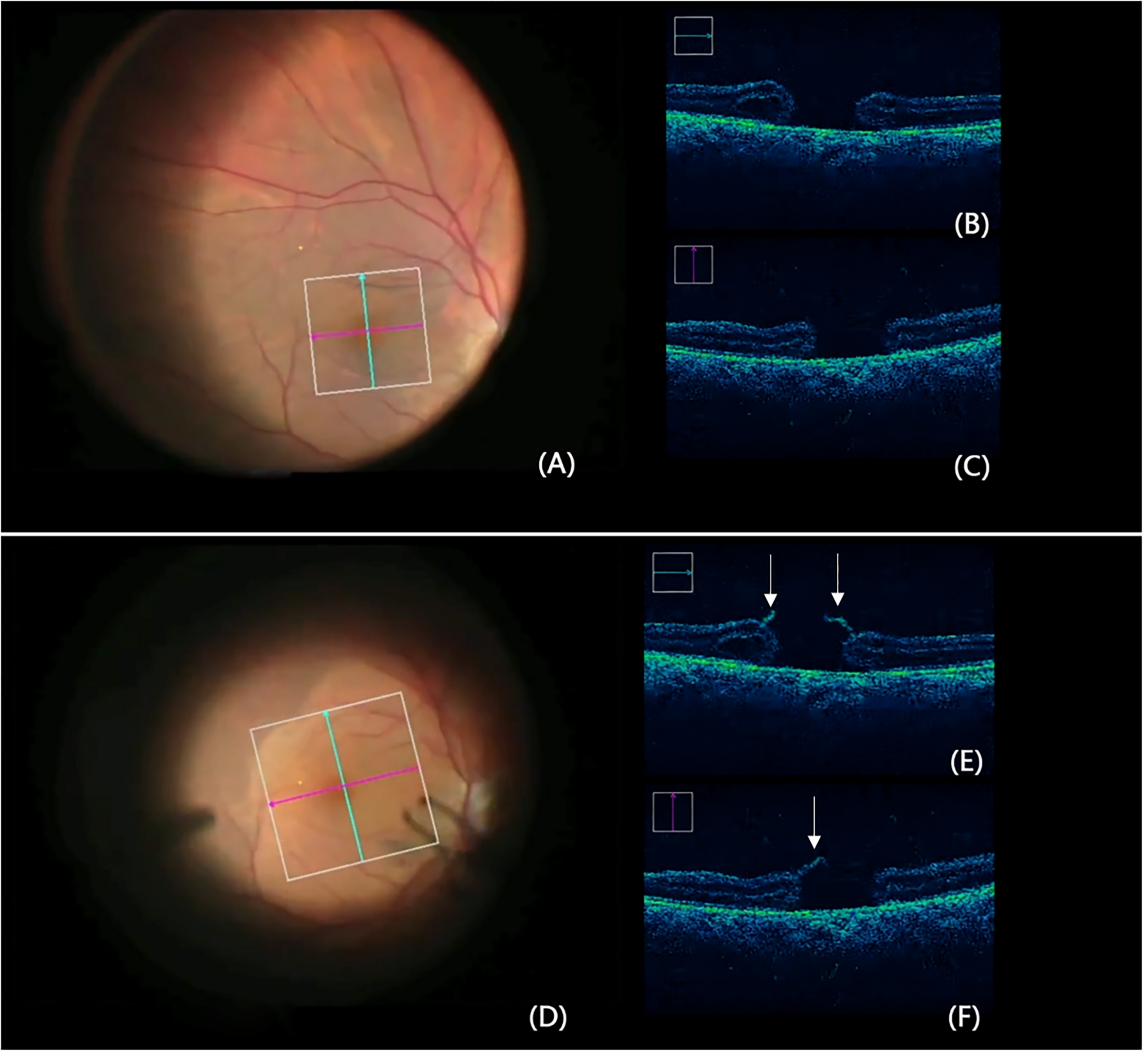
Synchronized iOCT imaging with the RESCAN 700 for Group identification. **(A–C)** Before ILM peeling: **(A)** Fundus view pre-peeling. **(B)** Horizontal and **(C)** vertical iOCT scans show the hole edges’ initial morphology. **(D–F)** After ILM Peeling: **(D)** Fundus view post-peeling. **(E)** Horizontal and **(F)** vertical iOCT scans display the “Hole-door” structure (white arrows), with vertical pillars of tissue extending into the vitreous cavity from the hole edges.

To ensure the precision of our grouping criteria, the real-time morphological changes of the hole edges were monitored using iOCT (RESCAN 700, ZEISS). The system provided synchronized visualization of the fundus image along with horizontal and vertical cross-sectional scans (Figure 1). This multi-planar imaging allowed for the definitive identification of “Hole-door” structures specifically following the release of tangential traction after ILM peeling (Figures 1E,F). These structures were either absent or less prominent in the pre-peeling stage (Figures 1B,C).

**FIGURE 2:**
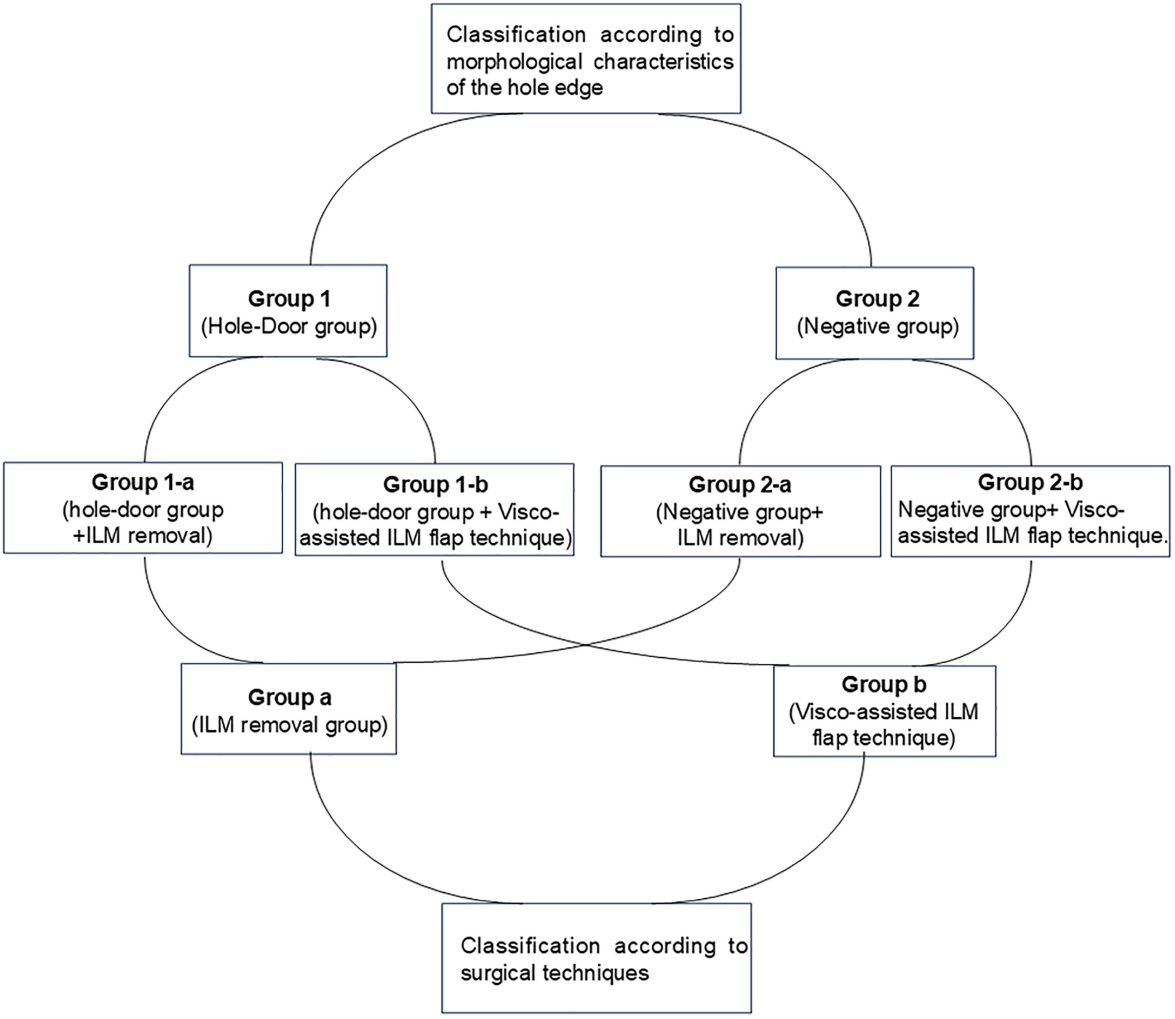
The specific grouping method.

Within each group, patients were further assigned to two subgroups according to the specific surgical intervention performed: complete ILM removal (Group 1-a and Group 2-a) or the visco-assisted ILM flap (Group 1-b and Group 2-b). To ensure balanced statistical power for subgroup comparisons, patients were consecutively enrolled according to strict inclusion and exclusion criteria until each of the four subgroups reached a sample size of 14 eyes, resulting in a total study population of 56 eyes. The specific grouping method is shown in Figure 2.

### 2.2 Surgical techniques

#### 2.2.1 Complete ILM removal

All procedures were performed using a 25-gauge pars plana vitrectomy under retrobulbar anesthesia by a single surgeon utilizing the Constellation Vision System (Alcon, United States). iOCT images were acquired using the RESCAN 700 scanner (ZEISS, Germany). Following the removal of the vitreous, the internal limiting membrane (ILM) was stained with 0.1% Indocyanine Green for 10 s. The ILM was then grasped with ILM forceps and peeled off over an area approximately equivalent to 2-disc diameters surrounding the macular hole (MH). An air-fluid exchange was subsequently performed with sterilized air. Finally, the cannula was removed, and the puncture site was ensured to be well closed without the need for suturing. Postoperatively, patients were advised to maintain a face-down position for a duration of 5 days.

#### 2.2.2 Visco-assisted ILM flap technique

During the peeling of the ILM around the MH, the ILM flap was not completely detached from the retina, instead, a remnant was intentionally left attached to the edges of the MH. The ILM flap measured approximately 1–1.5 diameter of the MH. The continuous infusion of basic salt solution (BSS) was closed, and an ocular viscoelastic device (OVD) was injected over the MH. The inverted ILM flaps were then carefully folded over the OVD-filled MH using forceps to minimize the risk of ILM flap dislocation. At the conclusion of the surgery, the eye was filled with sterilized air. Patients were advised to maintain a face-down position for a duration of 5 days.

### 2.3 Parameters assessed

The comprehensive demographic data, along with preoperative and postoperative best corrected visual acuity (BCVA), macular hole closure, and anatomical morphology of the photoreceptor layer extracted from optical coherence tomography (OCT) images (Heidelberg Engineering GmbH, Heidelberg, Germany) were collected. Photoreceptor layer restoration was assessed by reconstructing the continuous back reflection line for the EZ and ELM, defined as EZ (+) and ELM (+). Two independent observers evaluated the stored images, resolving disagreements by consensus. Clinical data and OCT scans from baseline and each follow-up visit (1 week, 3 and 6 months post-surgery) were retrieved from the hospital’s electronic database for analysis, which included BCVA measurement, slit-lamp examination, and OCT findings.

### 2.4 Statistical analysis

Statistical analysis was performed using IBM SPSS statistics (version 20.0) to analyze the differences in baseline characteristics, anatomical outcomes including macular hole closure, continuous restoration of the EZ and the ELM and functional outcomes of BCVA among four subgroups. The BCVA was recorded as decimal value and converted to LogMAR for statistical analysis.

The BCVA was compared using an unpaired *t*-test for parametric data and the Mann–Whitney test for non-parametric data. Changes within groups and subgroups before and after surgery were analyzed using the Wilcoxon signed-rank test. The closure of the MH and the continuous restoration of the EZ and ELM between groups and subgroups were assessed using the Chi-square test or Fisher’s exact test. A *P* < 0.05 was considered to be statistically significant.

## 3 Results

Fifty-six eyes of 56 patients (26 males, 30 females) were enrolled. The mean age was 58.79 ± 7.64 years (range 45–70), the average duration of macular hole is 3.46 ± 1.59 month (range 0.50–6.00), the average axial length is 23.76 ± 1.08 mm (range 22.00–25.98), the mean minimum hole diameter (MHD) was 542.89 ± 60.62μm (range 402–646), and the preoperative BCVA for all subjects was 1.00 ± 0.20 (range 0.7–1.5).

Based on the characteristics of the hole edge as determined by iOCT, the study categorized 28 eyes into the Hole-door group (Group 1) and another 28 eyes into the Negative group (Group 2). Within each group, 14 eyes underwent surgery with ILM removal (designated as Group 1-a and Group 2-a), while the remaining 14 eyes were treated using the Visco-assisted ILM flap technique (designated as Group 1-b and Group 2-b). At the baseline examination, no statistically significant differences were observed among the four subgroups in terms of age (*P* = 0.113), sex (*P* = 0.963), duration of macular hole (*P* = 0.310), axial length (*P* = 0.730), mean preoperative best-corrected visual acuity (BCVA) (*P* = 0.748), or MHD (*P* = 0.654). A comparison of the preoperative data across the four groups is presented in Table 1.

**TABLE 1:** Characteristics among four groups.

Characteristics	Groups				*P*-value
	Group 1-a (*N* = 14)	Group 1-b (*N* = 14)	Group 2-a (*N* = 14)	Group 2-b (*N* = 14)	
Gender (male/female)	6/8	7/7	6/8	7/7	0.963
Age (years)	55.57 ± 9.05	60 ± 6.74	57.21 ± 7.91	62.36 ± 5.30	0.113
Duration of MH (month)	3.64 ± 1.66	3.71 ± 1.64	3.71 ± 1.41	2.75 ± 1.61	0.310
Axial length	24.05 ± 1.08	23.74 ± 1.08	23.75 ± 1.22	23.53 ± 0.98	0.730
MHD (μm)	535.43 ± 70.49	560.29 ± 54.05	533.64 ± 56.86	542.21 ± 62.79	0.654
Preoperative BCVA	0.98 ± 0.21	0.99 ± 0.23	0.99 ± 0.15	1.04 ± 0.22	0.748

At the baseline examination, no statistically significant differences were observed among the four subgroups in terms of gender, age, duration of MH, axial length, MHD, or preoperative BCVA. Group 1-a, Hole-door group + ILM removal; Group 1-b, Hole-door group + Visco-assisted ILM flap technique; Group 2-a, Negative group + ILM removal; Group 2-b, Negative group + Visco-assisted ILM flap technique.

### 3.1 Correlation between MHD and surgical outcomes in large MHs

A correlation analysis was conducted to assess the potential impact of MHD on clinical outcomes. The analysis demonstrated no statistically significant correlation between preoperative MHD and any evaluated functional or anatomical outcomes (all *P* > 0.05). Specifically, there was no significant association between MHD and postoperative BCVA at either 3 months (*r* = –0.106, *P* = 0.437) or 6 months (*r* = –0.009, *P* = 0.949). Furthermore, preoperative MHD did not exhibit a significant correlation with the anatomical restoration of the ELM at 3 months (*r* = 0.025, *P* = 0.855) or 6 months (*r* = 0.113, *P* = 0.408), nor with the restoration of the EZ at 3 months (*r* = 0.006, *P* = 0.966) or 6 months (*r* = 0.055, *P* = 0.688).

### 3.2 Macular hole closure rates in four subgroups

The macular hole closure rates were 92.9% (13 out of 14) in Group 1-a, 100% (14 out of 14) in Group 1-b, 71.4% (10 out of 14) in Group 2-a, and 85.7% (12 out of 14) in Group 2-b. This difference was not statistically significant, with a *P*-value of 0.18.

### 3.3 Comparison of prognoses between the hole-door group and the negative group

#### 3.3.1 Comparison of visual acuity

**FIGURE 3:**
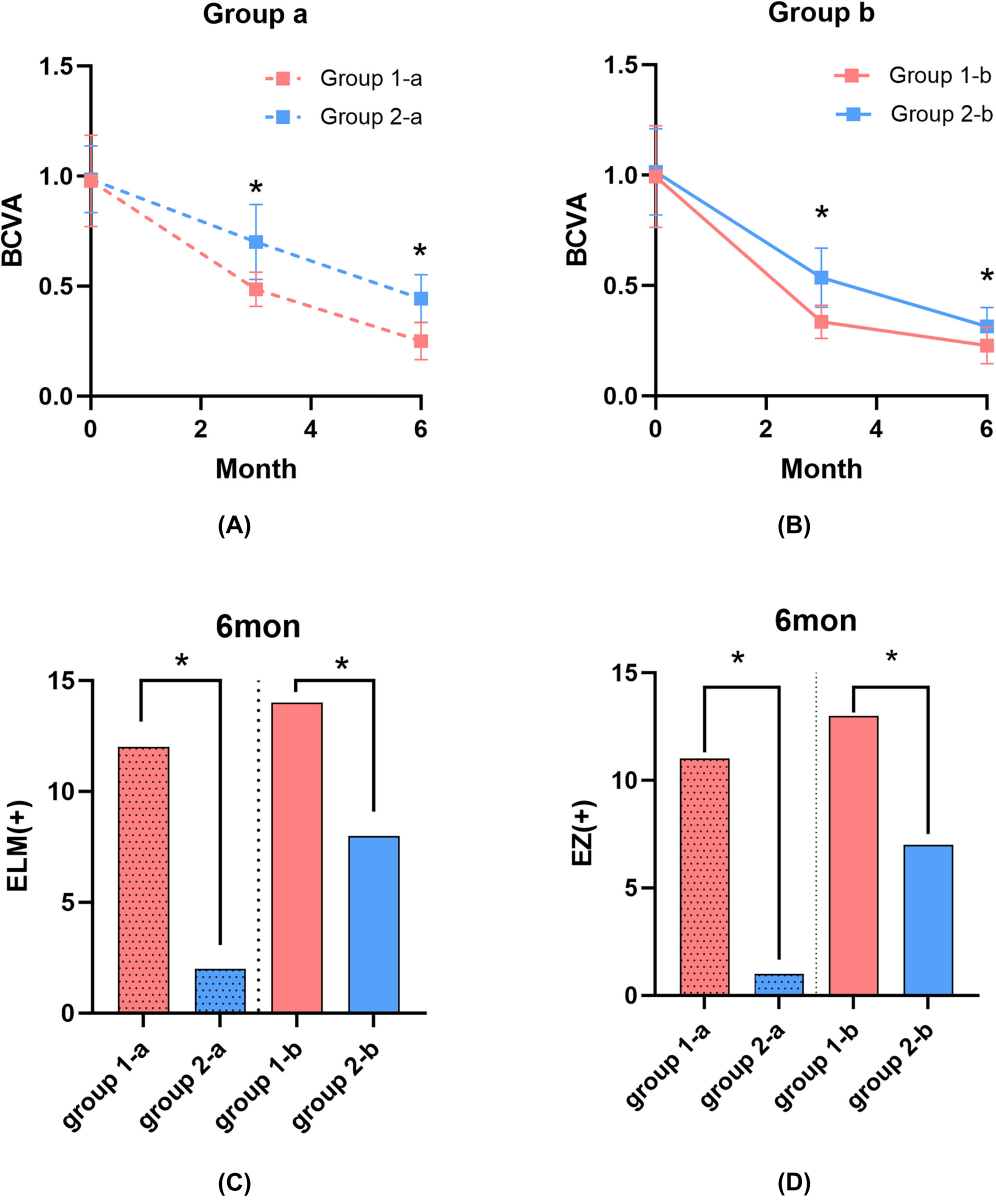
**(A)** Comparison of BCVA (LogMAR) between the Hole-door group and the Negative group at 3 and 6 months after ILM removal. **(B)** Comparison of BCVA (LogMAR) between the Hole-door group and the Negative group at 3 and 6 months after Visco-assisted ILM flap. **(C)** Comparison of ELM restoration between the Hole-door group and the Negative group at 6 months after ILM removal or Visco-assisted ILM flap. **(D)** Comparison of EZ restoration between the Hole-door group and the Negative group at 6 months after ILM removal or Visco-assisted ILM flap. *P < 0.05.

The Hole-door group had better visual acuity than the Negative group at 3 and 6 months postoperatively (*P* < 0.05), regardless of whether ILM removal or Visco-assisted ILM flap was used. In the ILM removal subgroups, Group 1-a had a mean BCVA of 0.49 ± 0.08 at 3 months and 0.25 ± 0.09 LogMAR at 6 months, compared to 0.70 ± 0.17 LogMAR and 0.44 ± 0.11 LogMAR in Group 2-a, respectively (Figure 3A). For the Visco-assisted ILM flap subgroups, Group 1-b had a mean BCVA of 0.34 ± 0.07 LogMAR at 3 months and 0.23 ± 0.08 LogMAR at 6 months, while Group 2-b had 0.54 ± 0.13 LogMAR and 0.31 ± 0.09 LogMAR, respectively (Figure 3B).

#### 3.3.2 Comparison of ELM and EZ recovery

Regardless of the procedure, the Hole-door group showed better ELM and EZ recovery than the Negative group at 6 months post-operative (*P* < 0.05). In the ILM removal subgroups, Group 1-a had 85.7% ELM (12 out of 14) and 78.6% EZ restoration (11 out of 14), while Group 2-a had 14.3% ELM (2 out of 14) and 7.1% EZ restoration (1 out of 14). In the Visco-assisted ILM flap subgroups, Group 1-b had 100% ELM restoration (14 out of 14) and 92.9% EZ restoration (13 out of 14), compared to Group 2-b’s 57.1% ELM (8 out of 14) and 50% EZ (7 out of 14) (Figures 3C,D).

Therefore, regardless of the procedure, the Hole-door group showed better recovery in vision, ELM, and EZ than the Negative group.

### 3.4 Comparison of prognoses between ILM removal and ILM flap for macular holes with or without Hole-door structures

#### 3.4.1 Comparison of visual acuity

**FIGURE 4:**
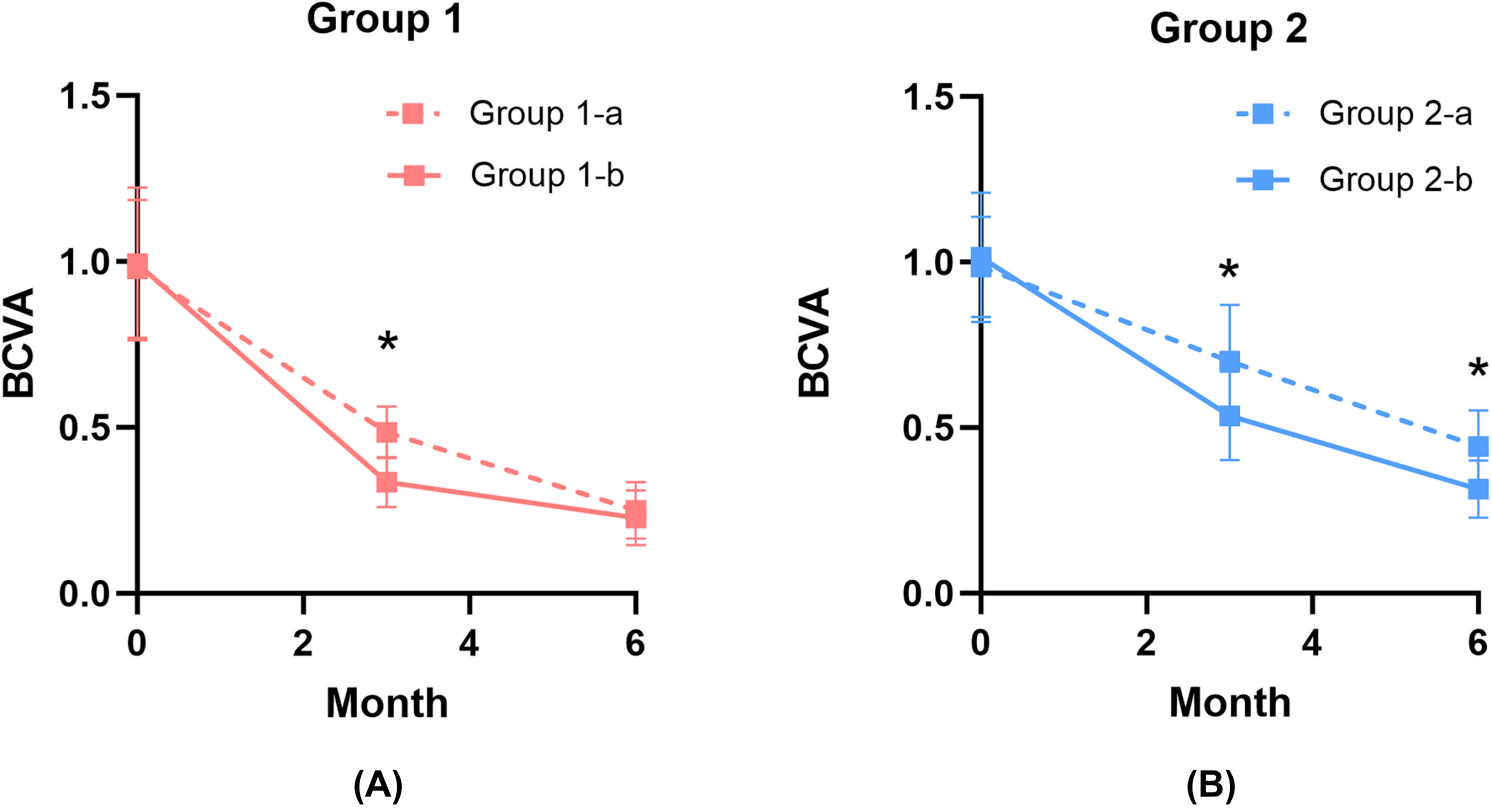
**(A)** BCVA (LogMAR) comparison between ILM removal and Visco-assisted ILM flap for the Hole-door group at 3 and 6 months. **(B)** BCVA (LogMAR) comparison between ILM removal and Visco-assisted ILM flap for the Negative group at 3 and 6 months. *P < 0.05.

In the Hole-door subgroups, Group 1-b had significantly better BCVA than Group 1-a 3 months after surgery (*P* < 0.05), with mean BCVA values of 0.34 ± 0.07 LogMAR and 0.49 ± 0.08 LogMAR, respectively. However, by 6 months, their BCVA differences were not significant (*P* > 0.05), with Group 1-a at 0.25 ± 0.09 LogMAR and Group 1-b at 0.23 ± 0.08 LogMAR (Figure 4A). In the negative subgroups, Group 2-b consistently outperformed Group 2-a in BCVA at both 3 and 6-months post-surgery (*P* < 0.05). At 3 months, Group 2-a had a mean BCVA of 0.7 ± 0.17 LogMAR, while Group 2-b had 0.54 ± 0.13 LogMAR. At 6 months, Group 2-a was at 0.44 ± 0.11 LogMAR, and Group 2-b at 0.31 ± 0.09 LogMAR (Figure 4B).

#### 3.4.2 Comparison of ELM and EZ recovery

**FIGURE 5:**
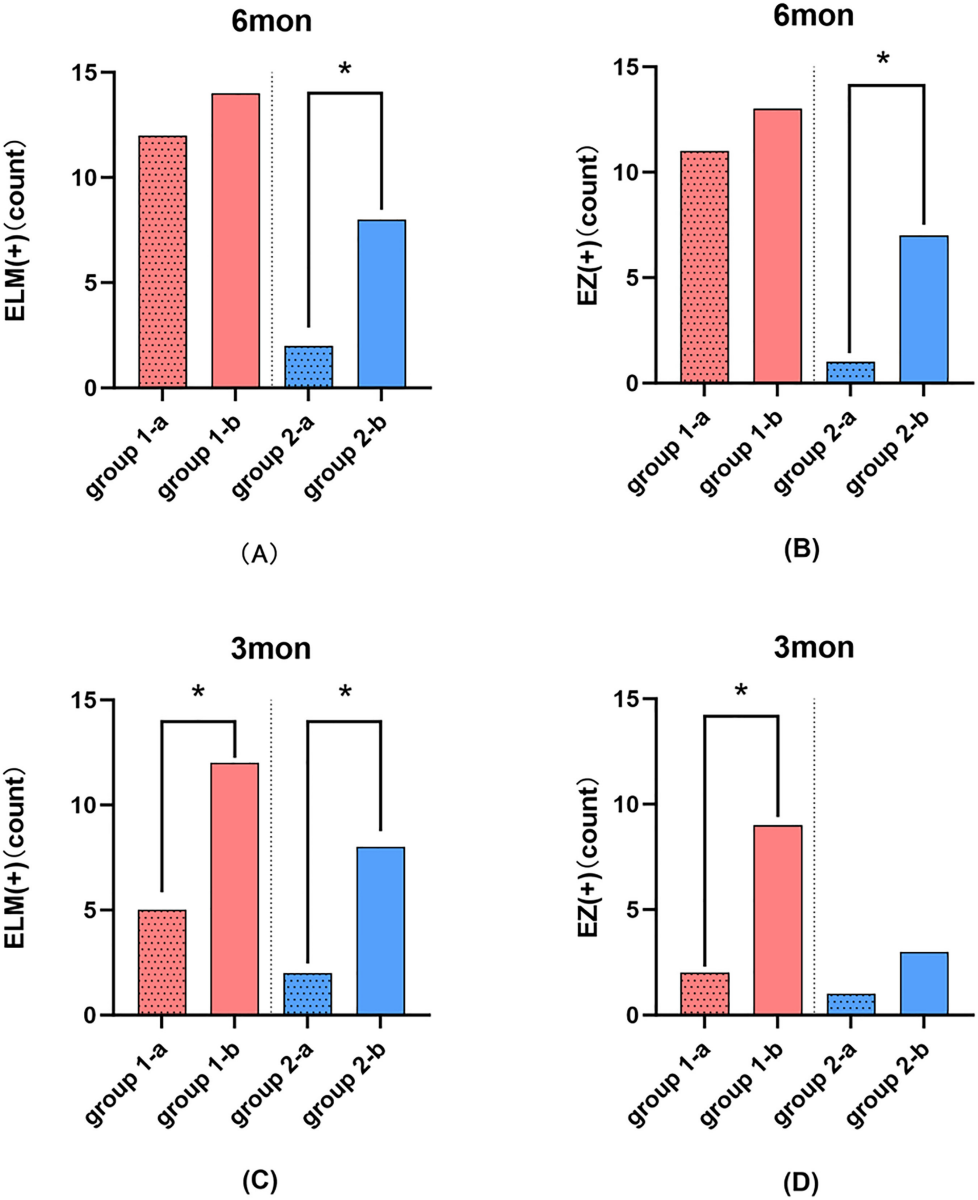
**(A)** Comparison of ELM restoration between ILM removal and Visco-assisted ILM flap for the Hole-door group and Negative group respectively at 6 months. **(B)** Comparison of EZ restoration between ILM removal and Visco-assisted ILM flap for the Hole-door group and Negative group respectively at 6 months. **(C)** Comparison of ELM restoration between ILM removal and Visco-assisted ILM flap for the Hole-door group and Negative group respectively at 3 months. **(D)** Comparison of EZ restoration between ILM removal and Visco-assisted ILM flap for the Hole-door group and Negative group respectively at 3 months. *P < 0.05.

At the 6-month follow-up, no significant differences were found in ELM or EZ restoration between Group 1-a and Group 1-b in the Hole-door subgroups. However, in the negative subgroups, Group 2-b showed significantly better restoration of ELM and EZ compared to Group 2-a. Specifically, Group 1-a had 85.7% ELM (12 out of 14) and 78.6% (11 out of 14) EZ restoration, while Group 1-b had 100% (14 out of 14) ELM and 92.9% (13 out of 14) EZ restoration. In contrast, Group 2-a had 14.3% (2 out of 14) ELM and 7.1% (1 out of 14) EZ restoration, whereas Group 2-b had 57.1% (8 out of 14) ELM and 50% (7 out of 14) EZ restoration (Figures 5A,B).

While, at 3 months after surgery, Group 1-b showed significantly better restoration of the ELM and EZ compared to Group 1-a, with ELM restoration rates of 85.7% (12 out of 14) versus 35.7% (5 out of 14) and EZ rates of 64.3% (9 out of 14) versus 14.3% (2 out of 14) (P < 0.05). In the negative subgroups, Group 2-b had significantly higher ELM restoration than Group 2-a (57.1% vs. 14.3%, *P* < 0.05), but no significant difference in EZ restoration, with rates of 21.4% (3 out of 14) for Group 2-b and 7.1% (1 out of 14) for Group 2-a (Figures 5C,D).

### 3.5 Correlation and consistency analysis between 3 and 6-month outcomes

**FIGURE 6:**
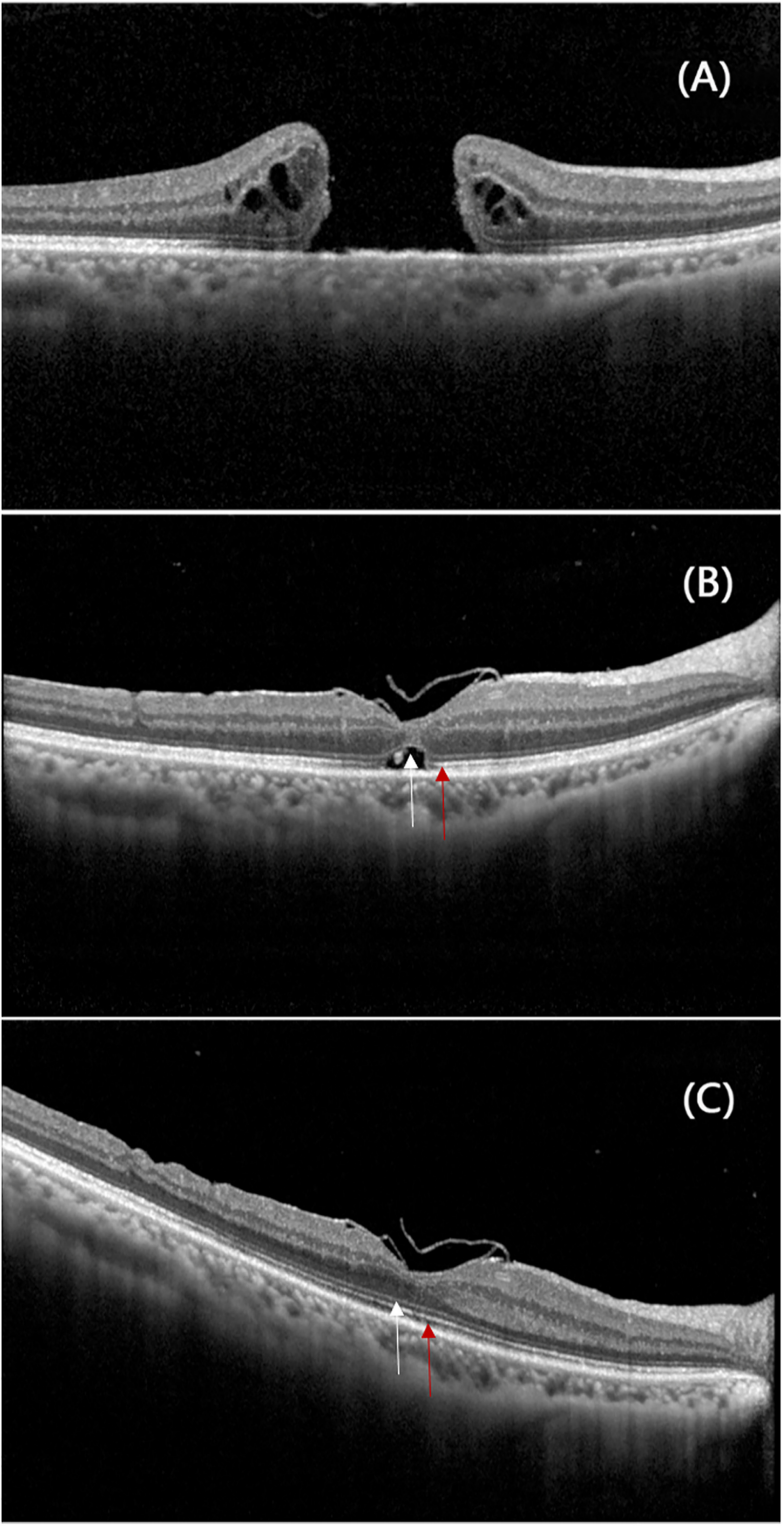
A 46-year-old man with a 515μm idiopathic macular hole was assessed via iOCT, revealing a distinctive Hole-door structure. He underwent visco-assisted ILM flap technique. **(A)** Preoperative imaging showed a full-thickness macular hole with disrupted outer retinal bands and visible discontinuities in the ELM and EZ at the edges. **(B)** Three months post-surgery, the macular hole was closed, the continuity of the ELM (white arrow) has been restored, though the EZ (red arrow) remained discontinuous. **(C)** By 6 months, both the ELM (white arrow) and EZ (red arrow) bands showed complete restoration.

Correlation and consistency analyses demonstrated that 3-month outcomes were significantly associated with 6-month results. Specifically, 3-month BCVA was strongly correlated with 6-month BCVA (*r* = 0.613, *P* < 0.001). Anatomical restoration also showed significant consistency (ELM: Kappa = 0.682; EZ: Kappa = 0.431; both *P* < 0.001). Notably, the McNemar test revealed a significant trend of continued microstructural reorganization between 3 and 6 months (*P* < 0.05) (Table 2), with the progressive restoration of the ELM and EZ layers clearly visualized in Figure 6.

**TABLE 2:** Correlation and consistency analysis between 3- and 6-month outcomes (*N* = 56).

Outcome	r or k	P (Spearman or Kappa)	P (McNemar)
BCVA (LogMAR)	0.613	<0.001	N/A
ELM restoration	0.682	<0.001	0.004
EZ restoration	0.431	<0.001	<0.001

Spearman’s rho (r) and Cohen’s Kappa (k) were used to assess functional and anatomical consistency between 3 and 6 months (all *P* < 0.001), while the McNemar test (*P* < 0.05) indicates significant continuous microstructural improvement during the follow-up period.

In summary, while there was no difference in visual acuity improvement and recovery of ELM and EZ at 6 months between ILM removal and ILM flap in the Hole-door group, the ILM flap showed significantly better outcomes at 3 months. This suggests that ILM flap can enhance visual acuity and ELM and EZ recovery in macular holes with Hole-door structures. The 3-month outcomes were reliable indicators of 6-month surgical success, showing significant correlation, while the restorative process continued to improve between 3 and 6 months. In addition, our analysis showed that those outcomes were independent of preoperative MHD but depended on the interaction between anatomical structure and surgical method.

## 4 Discussion

Recent studies have utilized iOCT to elucidate alterations in MH geometry after ILM removal (17, 18), identifying both Hole-door and foveal flap structures. Evidence suggests that Hole-door structures are significantly correlated with postoperative recovery of retinal microstructure and enhancement of visual function after ILM removal, particularly for larger macular holes. Moreover, the presence of Hole-door structures may serve as a predictive marker for superior postoperative visual recovery and restoration of ELM and EZ (16), which is consistent with some of our research findings. However, no studies have systematically compared the efficacy of various surgical techniques, such as ILM removal and ILM flap, in the treatment of macular holes with hole-door structures.

Our study investigated the outcomes of ILM removal and ILM flap surgeries in the treatment of large-diameter macular holes, both with and without Hole-door structures. The findings indicated that, irrespective of the presence of a Hole-door structure, the ILM flap technique was significantly more effective than ILM removal in improving BCVA and facilitating ELM and EZ recovery at the 3-month follow-up for both types of macular holes. These results align with numerous studies that have demonstrated the significant advantages of the ILM flap technique in managing large-diameter macular holes (19, 20). This method enhances adhesion at the hole edges by flipping the peeled ILM over the hole, thereby improving closure rates and postoperative visual recovery (21). A systematic review and meta-analysis have demonstrated that the ILM flap technique results in superior anatomical closure rates and enhanced ELM and EZ recovery across a range of macular hole sizes, with particularly significant efficacy observed in cases exceeding 400 micrometers (22, 23). In terms of functional recovery, the ILM flap technique presents distinct advantages. Evidence suggests that it outperforms ILM removal in achieving postoperative visual improvement, particularly in cases involving large-diameter macular holes (24).

The Hole-door structure is characterized by vertical tissue pillars that form along the edges of macular holes and are distinctly visible via iOCT. Kumar and Yadav (17) proposed that tissue pillars might consist of redundant retinal tissue, subclinical epiretinal membranes, or small ILM fragments at the hole’s edges. Therefore, the absence of this sign in some patients may be attributed to cleaner hole edges lacking such redundant tissue. Furthermore, the specific ILM peeling technique can significantly impact whether these pillars are successfully elevated and captured by iOCT (17).

Kumar and Yadav likened the closure mechanism to the inverted ILM flap technique, where these pillars offer mechanical support to bridge gaps and expedite defect coverage in the inner retina (25–27). In our study, macular holes lacking a Hole-door structure demonstrated superior postoperative BCVA and retinal microstructural recovery following ILM flap at a 6-month follow-up. In contrast, macular holes with a Hole-door structure exhibited no significant difference in BCVA and retinal microstructural recovery between ILM flap and ILM removal. However, our findings also indicated that the ILM flap technique further accelerated the recovery of BCVA, ELM, and EZ within the first 3 months in these patients. Although the precise mechanism remains to be fully elucidated, we hypothesize a synergistic effect where the endogenous “hole-door” pillars provide initial structural bridging, while the exogenous ILM flap serves as an additional biological scaffold that stabilizes the foveal microenvironment more rapidly. This combined support may facilitate earlier photoreceptor reorganization compared to ILM removal alone. Further research is required to confirm this hypothesis and explore the underlying biochemical or mechanical interactions between these two structures. Those proposed closure mechanism may elucidate the findings of our research which may offer a foundation for selecting appropriate clinical surgical methods.

The integrity of ELM and EZ is a well-established determinant of postoperative visual recovery (28). Quantitative spectral domain optical coherence tomography (SD-OCT) analyses have demonstrated that reduced foveal and parafoveal EZ defects are strong indicators of significant visual improvement (14). Furthermore, outer retinal reorganization is a gradual, time-dependent process, for instance, ELM restoration rates have been shown to increase from 40% at 1 month to 90% by 12 months postoperatively (13, 29). Importantly, early reconstruction of these layers serves as a robust predictor for subsequent anatomical and functional success (28, 30). Consistent with these findings, our study also demonstrated that 3-month functional and anatomical outcomes were highly predictive of 6-month results, while also observing significant further recovery of both the ELM and EZ during the follow-up period. In contemporary ophthalmic surgery, iOCT has emerged as an indispensable instrument for MH procedures (31, 32). This technology facilitates an objective evaluation of MH morphology and peripheral structures, such as the Hole-door architecture, which is essential for surgical decision-making and for predicting postoperative retinal microstructure recovery and functional outcomes (16). In this study, we retrospectively utilized iOCT data to examine the morphology of the Hole-door structure after ILM removal and to evaluate its impact on the selection of surgical strategies. Compared to the exclusive use of a microscope during the procedure, iOCT may provide more objective assessments, enhancing intraoperative decision-making.

To the best of our knowledge, this study is the first to compare visual acuity and ELM and EZ recovery after ILM removal and ILM flap for macular holes with Hole-door structures and revealed the guiding significance of the macular Hole-door structure in surgical strategy selection. In addition, there are several limitations to this study. First, the sample size was relatively small, with 14 eyes in each subgroup, which was constrained by the retrospective design and strict inclusion criteria during the study period. This may result in limited statistical power for certain parameters, such as the macular hole closure rate. However, significant findings were identified regarding postoperative BCVA and retinal microstructure restoration. Future multi-center studies with larger cohorts are needed to further validate these outcomes.

## 5 Conclusion

Macular holes with Hole-door structures exhibit a more favorable prognosis compared to those without such structures. Furthermore, among macular holes with Hole-door structures, no significant prognostic difference was noted between the two surgical techniques, namely ILM removal and ILM flap. Nonetheless, ILM flap technique was observed to accelerate the improvement of BCVA, as well as the restoration of the ELM and EZ, in cases of macular holes with Hole-door structures.

## Data Availability

The raw data supporting the conclusions of this article will be made available by the authors, without undue reservation.
